# Evaluating geographic accessibility to COVID-19 vaccination across 54 countries/regions

**DOI:** 10.1136/bmjgh-2024-017761

**Published:** 2025-02-19

**Authors:** Yanjia Cao, Tianyu Li, Huanfa Chen, Qunshan Zhao, Jiashuo Sun, Karen Ann Grépin, Jeon-Young Kang

**Affiliations:** 1 Department of Geography, The University of Hong Kong, Hong Kong, Hong Kong SAR; 2 UCL Centre for Advanced Spatial Analysis, London, UK; 3 Urban Big Data Centre, School of Social & Political Sciences, University of Glasgow, Glasgow, UK; 4 School of Public Health, University of Hong Kong Li Ka Shing Faculty of Medicine, Hong Kong, Hong Kong SAR; 5 Department of Geography, Kyung Hee University, Seoul, Republic of Korea

**Keywords:** COVID-19, Geographic information systems, Health Services Accessibility, Global Health

## Abstract

**Background:**

The COVID-19 pandemic has revealed significant disparities in global vaccine accessibility, particularly affecting low and middle-income countries (LMICs). However, current research on COVID-19 vaccine accessibility primarily focuses on individual countries or high-income countries (HIC). We aimed to evaluate geographic accessibility to COVID-19 vaccination on a multicountry scale, covering comparisons across LMICs and HICs. Additionally, we explored the potential economic factors related to accessibility and their impacts on health outcomes.

**Methods:**

We collected population data at a 1 km resolution and geocoded all vaccination sites across the selected countries/regions. Four measures were used to evaluate vaccine accessibility from different perspectives: population coverage with varying travel time thresholds, driving time to vaccination sites, the number of sites within a 30-min threshold and a geographic accessibility index using enhanced two-step floating catchment area method. Finally, we explored the relationships between geographic accessibility and several factors: gross domestic product per capita, vaccination uptake and mortality.

**Findings:**

We found substantial disparities in vaccine accessibility across the selected countries/regions. In 24.07% of these countries/regions, over 95% of the population can access the nearest vaccination services within 15 min. In contrast, in countries/regions such as Manitoba (Canada), Zimbabwe and Bhutan, less than 30% of the population can reach these sites within 60 min. Underserved areas, termed ‘vaccine deserts’, were identified in both HICs and LMICs. We found that countries/regions with higher vaccine accessibility tend to achieve higher vaccination rates, whereas those with lower vaccine accessibility are likely to experience substantial increases in COVID-19 mortality rates.

**Conclusion:**

LMICs require enhanced attention to improve geographic accessibility to vaccination. Additionally, there are internal disparities in accessibility within both HICs and LMICs. National public health officials and global health initiatives are suggested to prioritize ‘vaccine deserts’ and to ensure equitable vaccine access in future pandemics.

**WHAT IS ALREADY KNOWN ON THIS TOPIC**

**WHAT THIS STUDY ADDS**

- To our knowledge, this study is the first to investigate and compare geographic accessibility to COVID-19 vaccination at the subnational level across multiple countries/regions. It enriches the existing evidence by clearly highlighting the disparities in vaccine accessibility both within and between low and middle-income countries and HICs. Additionally, we discussed vaccine accessibility in relation to the local economy status as well as rates of vaccination and COVID-19 mortality on 1 December 2020 and 30 June 2022. This provides new insights into the effectiveness of COVID-19 control strategies.

**HOW THIS STUDY MIGHT AFFECT RESEARCH, PRACTICE OR POLICY**

- This research enables countries/regions to compare their vaccine accessibility performance using various evaluation criteria and to identify underlying reasons for weaknesses through comparison with similar nations. Insights from this research potentially guide public health officials to identify ‘vaccine deserts’ caused by transportation barriers, limited vaccine supplies with the higher vaccine demands and lower socioeconomic status. By prioritising vaccine allocation in high-risk or underserved areas, they can boost vaccination uptake among residents and thereby reduce the disease burden.

## Introduction

 Since December 2019, the COVID-19 pandemic was responsible for a devastating number of infections and deaths worldwide. This crisis had unprecedented health, social and economic challenges.^1^ In the early stages of the pandemic, public health officials worldwide primarily adopted non-pharmaceutical interventions, such as social distancing, to reduce transmission and mitigate the impact of the virus. By December 2022, at least 50 types of vaccines had been approved by one or more countries, and 201 countries had authorised at least one COVID-19 vaccine,^2^ marking a new phase where vaccines could play important and complementary roles in government pandemic response plans.

Building on insights from the 2009 H1N1 pandemic,^3^ the WHO established a global vaccine distribution programme called COVID-19 Vaccine Global Access (COVAX), in collaboration with other global health institutions, such as Gavi.^4 5^ This programme explicitly emphasised that no country should vaccinate more than 20% of its population until every country has reached this goal.^6^ The partnership worked together to ensure equitable vaccine access, especially in low and middle-income countries (LMICs). However, this global cooperation initiative still faced numerous challenges in its operation, resulting in COVID-19 vaccination rates highly unequal by 2023.^7^ The primary reasons for persistent vaccine inequality include limited economic support in LMICs, vaccine hesitancy and limited accessibility to vaccine services in these countries.^8 9^ For example, many high-income countries (HICs) chose not to purchase vaccines through COVAX. Instead, they secured premium access to large quantities of COVID-19 vaccines by prioritising bilateral deals with pharmaceutical companies, posing a risk of vaccine shortages for COVAX.^10^ As a result, HICs purchased and stockpiled far more vaccines than their residents needed, while LMICs could not secure enough to meet their demands.^11 12^ Evidence indicates that, on average, LMICs started their COVID-19 vaccinations 100 days later than HICs.^13^ Beyond vaccine supply and distribution, variations in infrastructure and attitudes towards vaccination also played a role. Compared with HICs, LMICs often suffered from greater structural barriers to vaccination, such as vaccine logistics and inaccessible locations.^14^ According to a report by the Gavi Vaccine Alliance, approximately 20% of countries could not afford cold-chain facilities, making it challenging to store cold-chain vaccines properly.^15^ In addition, individuals in LMICs had to travel long distances to vaccination sites or encountered obstacles due to political crises.^14 16 17^

Notably, research has demonstrated a correlation between vaccine accessibility and vaccination rates. Studies have found that a higher density of vaccine sites correlates with increased vaccination rates across US counties, regardless of socioeconomic status.^18^ Another study at US County level indicated that adding a vaccination site via retail pharmacy boosted per-capita vaccine doses by approximately 26%.^19^ The study also revealed that one additional pharmacy per 10 000 people reduced weekly COVID-19 deaths by 2.7%.^19^ When comparing across countries, researchers focused on exploring the heterogeneity in COVID-19 vaccine coverage and associated factors from a global perspective. For example, a study on 194 countries revealed that countries with higher socioeconomic characteristics and advanced healthcare resources achieved higher vaccine coverage.^20^ From a geospatial resource planning perspective, geographic information systems and spatial analysis tools provide technical support for identifying service coverage gaps. With this comprehensive approach, researchers are able to explore regional health inequities and develop evidence-based allocation strategies.^21 22^ One case study in Chicago illustrated the application of geographic analysis in optimising vaccine allocation strategies through identifying 15 vulnerable communities that required targeted intervention.^23^ The study also revealed spatial variations in vaccination rates, indicating that standardised policy implementation produces different outcomes across regions. The evidence suggests that effective strategies on vaccine allocation should incorporate vaccination accessibility analysis within the community context.^23^ While existing research has established various analytical frameworks for vaccine accessibility, most studies focused on individual countries, particularly in HICs. One study in the USA identified ‘vaccine deserts (ie, regions with geographic obstacles to vaccine-related herd immunity)’ mainly in rural and medically underserved areas.^22^ Another study in England reported that areas with lower deprivation and higher percentages of black populations tend to have lower vaccination rates.^24^ However, the lack of studies in LMICs and related comparative work limited the findings of global inequalities in vaccine accessibility. Since disease transmission is not bounded by national borders, the inequalities in disease control between LMICs and HICs are likely to exacerbate the pandemic.^25^ As a result, this situation potentially extended the pandemic, as evidenced by the outbreak of Omicron,^26 27^ thus requiring efforts from all countries to address both the urgent health crisis and the economic burden from healthcare costs.^25^ Despite the importance of investigating vaccine accessibility, there remains a knowledge gap in understanding the heterogeneity of COVID-19 vaccine accessibility across income levels, particularly between HICs and LMICs.

This study introduced an innovative framework to evaluate geographic accessibility to COVID-19 vaccination sites across 54 countries/regions. Integrating vaccine supply, population demand and travel time impedance, we explored the accessibility at 1 km resolution using multiple metrics. Furthermore, we investigated the economic factors that contribute to disparities in vaccine accessibility and explored their potential relationships with vaccination rates and COVID-19 mortality outcomes across various stages of the pandemic. An important contribution of this research is that we expanded the scope of analysis to both HICs and LMICs and applied a common framework to both areas. To our knowledge, this is the first study to comprehensively investigate vaccine accessibility and corresponding health outcomes across countries with diverse economic backgrounds. As a retrospective study for the COVID-19 pandemic, our multicountry analysis identified regions where limited access exacerbated low vaccination rates and higher mortality outcomes. We recommend global health institutions take action to optimise resource allocation. For each country/region, our comparative analysis helps public health officials better understand vaccine performance and develop effective intervention plans for future pandemics.

## Methods

### Study design and data sources

**Figure 1:**
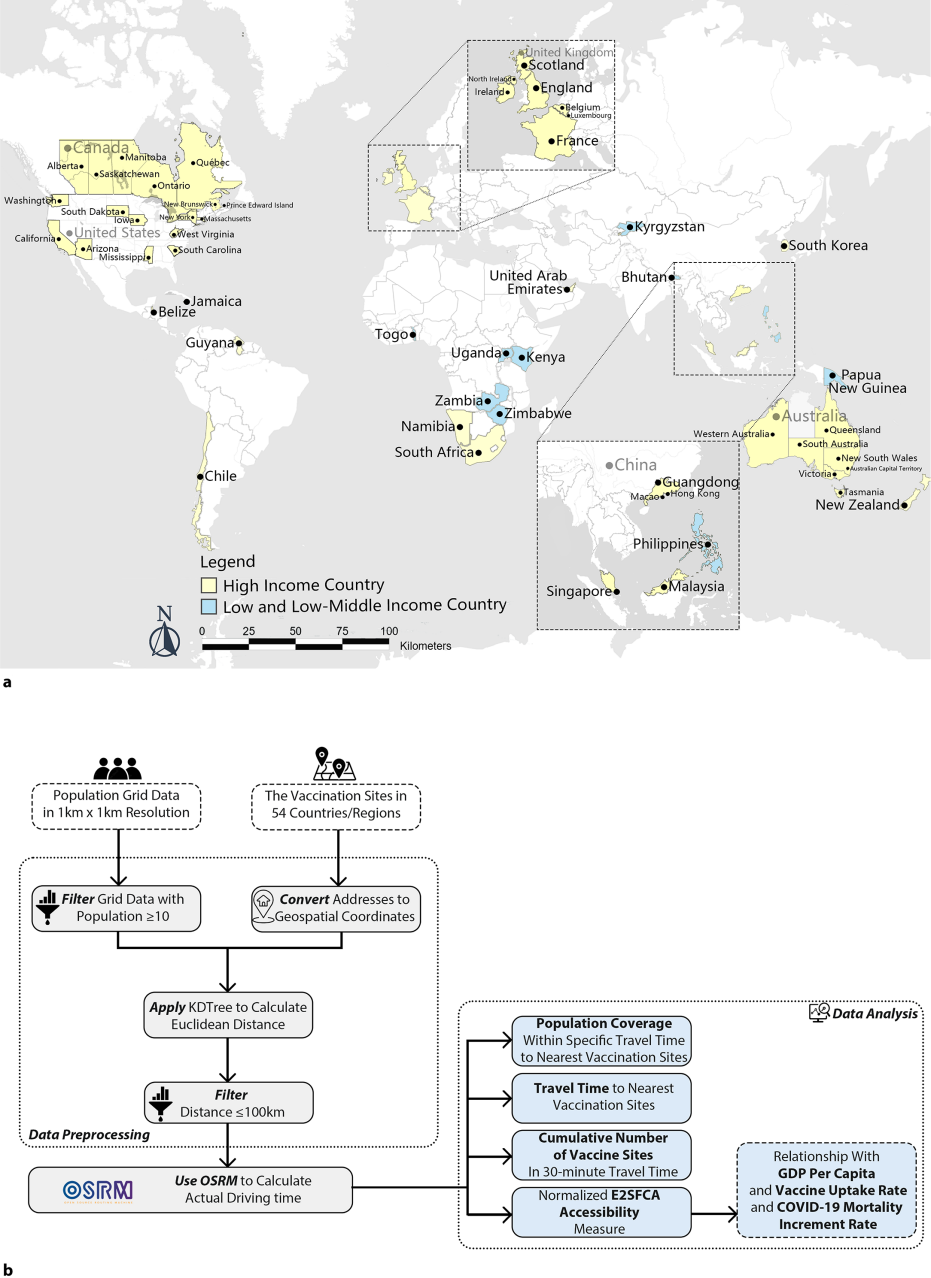
(**a**) The geographic location of the selected countries/regions; (**b**) research framework. E2SFCA, Enhanced Two-Step Floating Catchment Area.

The study selected 54 countries/regions across six continents, including nine from LMICs, as illustrated in figure 1a. The selection criteria are primarily based on the availability of data from these countries/regions. We also selected 30 subnational regions out of five countries based on two criteria: (1) the selected regions are comparable in both areas and populations; (2) each region has an independent public health management system. Such criteria also made these subnational regions comparable with other national level data. The regions included the provinces of Canada, the states in the USA and Australia, as well as regions in the United Kingdom (UK), and two special administrative regions in China. Although the sample regions were limited by the data sources, this research aims to introduce our evaluation framework for vaccine accessibility in as many places as possible during the COVID-19 pandemic and provide as much data as we can to benchmark and rank areas with available data.

We retrieved country/region population data from WorldPop at a 1 km resolution for 2020 (online supplemental source 1). Vaccination sites were mainly sourced from the health department website of each country (online supplemental source 2). These data were collected during August 2021 and September 2023. We used street network from OpenStreetMap (OSM) to calculate geographic accessibility. To further discuss its relationship with the country’s economic development, we obtained 2022 country-level Gross Domestic Product (GDP) per capita data (USD) from the World Bank.^28^ For regional data presented in local currencies on their government websites (eg, Canada, Australia and the UK), we converted them to US dollars using the exchange rate (online supplemental source 3 and table S1). We further explored two specific health metrics: individuals fully vaccinated per hundred people and COVID-19 deaths per million population. We collected data from three critical phases of the COVID-19 response: 1 December 2020, marking the initial vaccine introduction; 1 May 2021, during the mid-stage of vaccine deployment; and 30 June 2022, in the late-pandemic phase. The country-level data were all collected from WHO (online supplemental sources 4–6). State-level data were retrieved from the national health data platforms of the USA, UK, Australia and Canada (online supplemental sources 4–6).

Following the research framework displayed in figure 1b, this study evaluates geographic accessibility to COVID-19 vaccination on a multicountry scale, encompassing comparisons across both LMICs and HICs. Utilising data with a 1 km resolution and geocoded vaccination sites, we evaluated vaccine accessibility from different perspectives: (1) population coverage within 15 min, 30 min and 60 min driving time thresholds of the nearest vaccination sites, (2) the patterns of driving time to the nearest vaccination site, (3) cumulative number of vaccination sites within 30 min and (4) a geographic accessibility index using enhanced two-step floating catchment area (E2SFCA) method. Additionally, we explored the relationships between the geographic accessibility index and several factors: (1) GDP per capita, (2) vaccination rates and (3) COVID-19 mortality increment. These relationships were evaluated through both exploratory analyses and log-linear regression models.

### Geocoding vaccination sites

The original vaccination site data only provided street addresses with postal codes, such as ‘National Road, Población, 8M8V+PGC, Luba, 2813 Abra, Philippines’. We first geocoded address information to geographic coordinates (longitude and latitude) using the WGS-84 coordinate system through the Google Maps Geocoding API (Hypertext Transfer Protocol, Application Programming Interface, see online supplemental method S1).^29^ We then converted population data from 1 km^2^ grids to points in ArcGIS Pro, adding centroid latitude and longitude to represent geographic location. We only retained grids with a population over 10 to protect health-related privacy.

### Driving time estimates for vaccine site access

To evaluate geographic accessibility to COVID-19 vaccines, we first constructed an origin–destination (OD) matrix between each population centroid and vaccination site. To further reduce computational complexity, we excluded OD pairs over 100 km apart in Euclidean distance, using the K-Dimensional Tree (KD-tree) method.^30^ As many COVID-19 vaccination sites are primarily situated in car-accessible areas,^31^ we used the Open-Source Routing Machine (OSRM) specifically for calculating driving times between each pair of filtered population centroids and vaccination sites. OSRM is a high-performance open-source routing engine designed to employ OSM data via its HTTP API. Using OSRM, we aligned OD points to the nearby street network, identified the fastest route between OD points and calculated the shortest travel time.^32^ OSRM also applied corresponding speed limits for different types of roads (online supplemental method S2).

### Four metrics for geographic accessibility computation

Geographic accessibility refers to the degree of effort required to reach medical services.^33^ In this study, we examine accessibility to vaccination services through four different metrics: population demand, travel time impedance, vaccine availability and a comprehensive evaluation. These indicators construct a multidimensional framework that captures both spatial and temporal aspects of vaccine accessibility, thus augmenting the understanding of service distribution and potential access barriers.

First, vaccine population coverage was determined through the percentage of the country/region population within 15 min, 30 min and 60 min of driving time to the nearest vaccination sites, respectively. This indicator offers clear benchmarks across countries/regions for policy evaluation. Then we estimated travel time patterns by visualising probability density function.^34^ The density histogram approach standardised the comparison of accessibility patterns across regions by calculating travel times from each population grid centroid to the nearest vaccination site. We also applied kernel density estimation to create a continuous probability density estimate.^34^ This non-parametric approach balanced the limitations using histogram binning and effectively captured irregular distributions of travel times.

Furthermore, we used the cumulative number of vaccination sites to further compare vaccine resources across countries/regions.^34^ This metric quantifies the spatial concentration of vaccination services, indicating service density in different regions.^35^ According to WHO guidelines, district healthcare facilities should ideally be within 15–30 min of driving from their communities.^36^ Therefore, we defined a 30 min threshold to sum up the cumulative sites for each 1 km^2^ population grid.

Finally, to integrate vaccination service distribution, travel time impedance and population demand, we applied an E2SFCA method^37^ to provide a comprehensive understanding of geographic accessibility across countries/regions. The metric is expressed as follows:

(1) $$R_{j}=\frac{S_{j}}{\sum_{i}D_{i}w(t_{ij})}$$

where $S_{j}$ refers to the vaccine capacity at vaccine site j. In this research, we assumed equal availability of vaccination across sites and thus *S_j_*=1. *D_i_* indicates the population grid i. $t_{ij}$ denotes the travel time between population grid i and vaccine supply point *j.w* (*t_ij_*) is the distance decay function of *t_ij_*. We used the Gaussian function $w(t_{ij})=e^{\frac{-t_{ij}^{2}}{\beta }}$ to represent the decay of vaccine accessibility as travel time increases. The catchment of vaccine site j is defined as the area within a 30 min driving zone. The value of β was determined when $t_{ij}=30$ and *w* (*t_ij_*) approach 0. In the second step, the accessibility index $A_{i}$ is calculated as follows:

(2) $$A_{i}=\sum_{j}R_{j}w(t_{ij})$$

We aggregated the weighted supply-to-demand ratios $R_{j}$ across all vaccine sites within the designated service area of population grid i. The weighting is again determined by the Gaussian distance decay function.

The computations with 1 km^2^ population grids were combined for all countries/regions. We normalised all scores on a scale between 0 and 1 and categorised the values into quintiles. Correspondingly, the categories are defined in ascending order: very low accessibility for the lowest 20%, low accessibility for 20%–40%, moderate accessibility for 40%–60%, high accessibility for the highest 20%–40% and very high accessibility for the highest 20%. The analysis and visualisation were conducted by Python V.3.9.11 and ArcGIS Pro 3.0.

### Correlation analysis between vaccine accessibility and associated factors

To further explore the relationships between vaccine accessibility and GDP per capita, vaccination rates and increase in COVID-19 mortality per million people, respectively, we conducted our analysis in two steps. We first used scatter plots for visual exploration of data distribution. Then, we performed log-linear regression analysis. The vaccine accessibility was calculated as the mean value of E2SFCA scores of population grids for each country/region. Since skewed distributions are commonly found in economic and ratio-based data, we applied log transformation to normalise the distributions and linearise potential exponential relationships.^38^ We then built three log-linear regression models to investigate the associations between vaccine accessibility and these factors. Each model was visualised through scatter plots with fitted lines, including 95% CIs, $R^{2}$ and p values.

## Results

### Population coverage with different travel time thresholds

**Figure 2:**
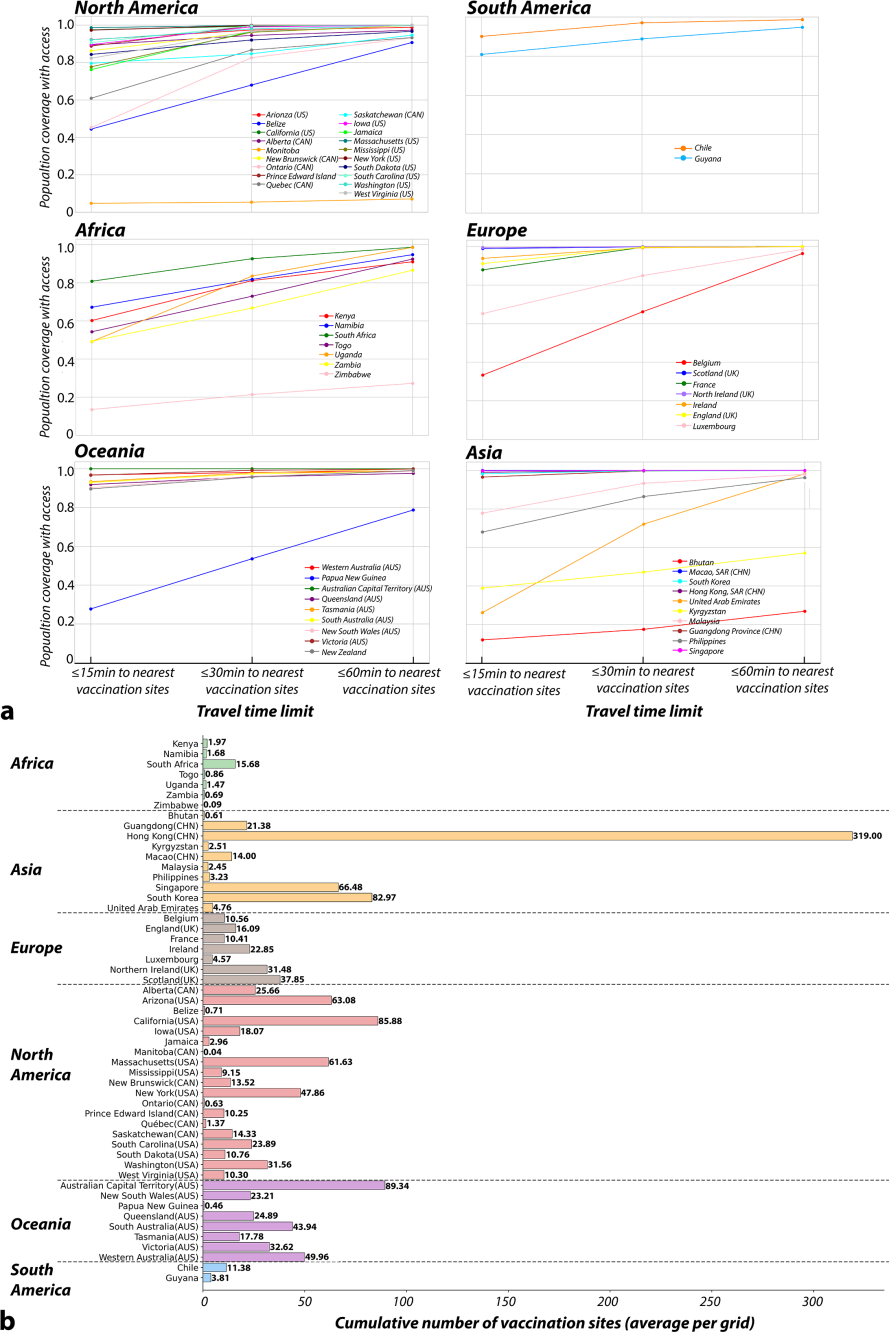
(**a**) Population coverage by vaccination within 15 min, 30 min and 60 min driving time. (**b**) Average number of vaccination sites within 30 min across 1 km² grids of each country/region.

At the country/region level, population coverage of vaccination sites showed substantial differences in travel time thresholds (ie, driving time), as illustrated in figure 2a. In 24% of the countries/regions, over 95% of the population were covered within a 15 min travel time. The number of countries/regions increased to 61% with the 30 min driving time threshold. In four countries/regions (ie, Manitoba (CAN), Zimbabwe, Bhutan, Kyrgyzstan), more than half of the population reside beyond 30 min travel to the nearest vaccination site. Particularly in Manitoba (CAN), Zimbabwe and Bhutan, over 70% of the population needed to travel over 60 min to receive the nearest vaccination site, highlighting severe concerns with vaccine accessibility.

Intercontinental disparities in population coverage were also evident. Taking a 15-min time interval as an example: in North America, 36% of countries/regions had achieved over 90% population coverage for vaccines. Meanwhile, in Europe, Scotland and England had reached nearly 99% vaccine coverage, while Belgium lagged with only 33% coverage. In Oceania, most selected countries/regions achieved over 90% coverage; however, Papua New Guinea was a notable exception with only 28% coverage. In Asia, Bhutan, United Arab Emirates (UAE) and Kyrgyzstan reported lower coverage of 12%, 26% and 39%, respectively. African countries, except for South Africa, required urgent attention due to their limited population coverage compared with other global nations. In these countries, less than 70% of the population was covered within 15 min. Detailed population coverage for each country/region is found in online supplemental table S2.

### Cumulative number of vaccination sites within 30 min

The cumulative number of vaccination sites within 30-min travel thresholds also showed substantial disparities at the country/region level, as illustrated in figure 2b. Among the 54 countries/regions analysed, only 26% had more than 30 vaccination sites, in stark contrast to the 22% where fewer than two sites were available. The highest number was in Hong Kong SAR at 319, while the lowest was in Manitoba (CAN) at 0.04.

In Oceania, Europe and North America, the cumulative number of vaccination sites generally exceeded 10, indicating that populations in these countries/regions typically had access to more than 10 vaccination sites within a 30-min travel time. In Asia, countries/regions with high levels of urbanisation such as Hong Kong SAR (cumulative number N=319), South Korea (N=83) and Guangdong Province (N=21) reported a high volume of vaccination sites. However, in most Southeast and Central Asian countries such as Malaysia (N=2), the Philippines (N=3) and Bhutan (N=1), the numbers were considerably lower. The situation is particularly concerning in Africa. All selected African countries, except South Africa (N=16), had less than two sites within 30 min travel time. The issue was particularly severe in Zimbabwe, Zambia and Togo, with almost no vaccination sites accessible (N<1).

### Travel time to the nearest vaccination site

**Figure 3:**
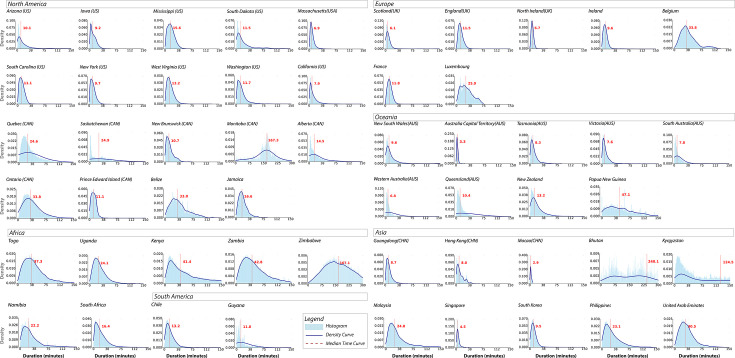
Travel time to the nearest vaccination site in selected countries/regions.

Among the 54 countries/regions, 59.26% reported a median travel time (ie, driving time) of 15 min or less to the nearest vaccination site, as displayed in figure 3. However, travel times varied among these countries/regions. Four of them—Manitoba (CAN), Zimbabwe, Bhutan and Kyrgyzstan—recorded median travel times exceeding 60 min, with Bhutan experiencing the longest at 240.15 min.

Countries/regions that exhibited similar median travel times to the nearest vaccination site demonstrated similar travel time distribution. For instance, in countries/regions where the median travel time was within 15 min, including all the selected states in the USA, the distribution was either right skewed or approximately normal. However, some countries/regions, such as Saskatchewan (CAN, median time=24.87 min), Queensland (AUS, 10.43 min) and Bhutan (240.15 min), were exceptions. Their travel time patterns exhibited lower peaks, broader distributions and greater variations without a cluster. Details of each country/region are found in online supplemental table S2.

### Global comparison of vaccine accessibility levels

**Figure 4:**
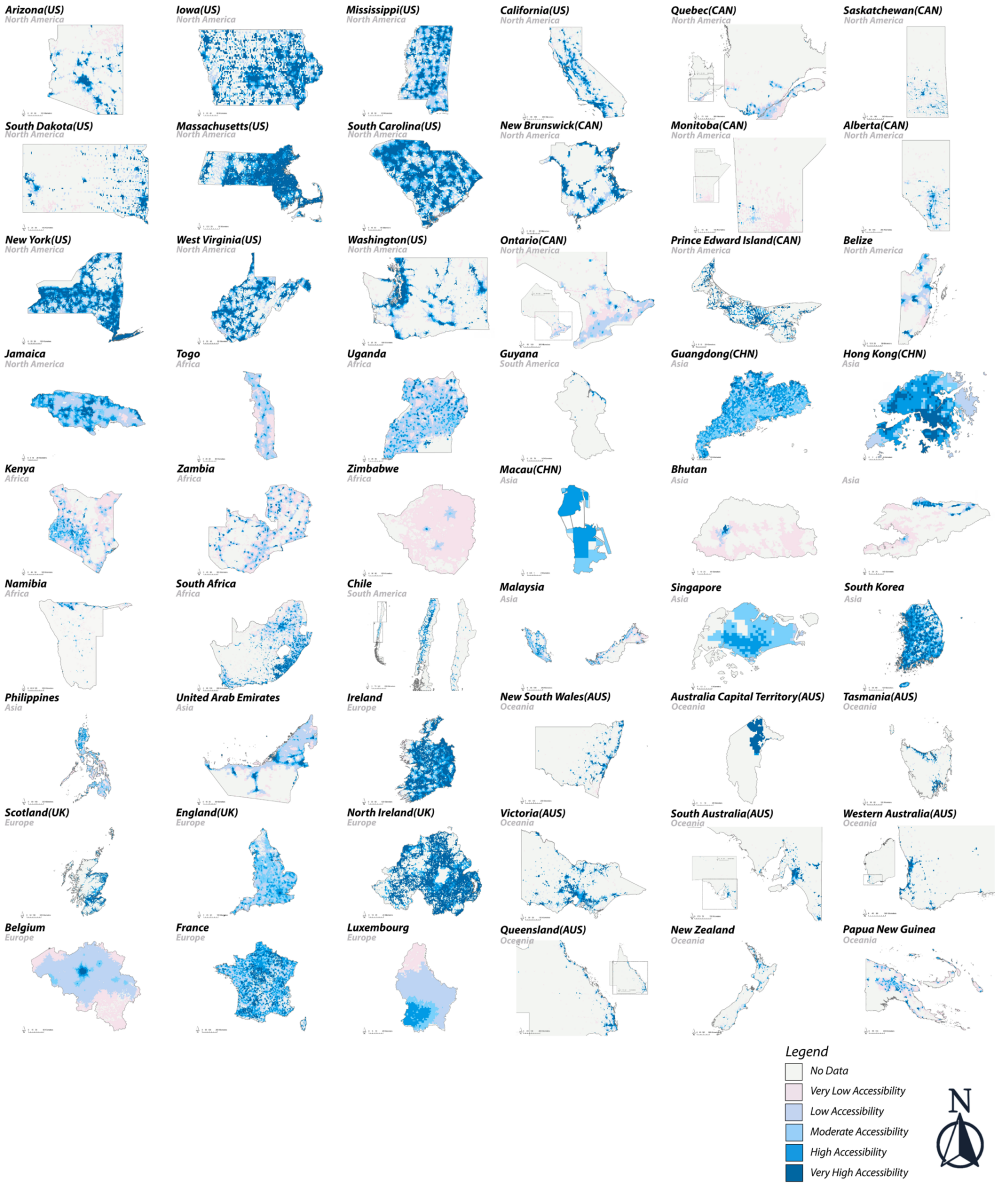
Comparison of vaccine accessibility levels in selected countries/regions.

Using the E2SFCA method, our multicountry analysis revealed considerable disparities in vaccine accessibility across both low and high population density countries/regions, as displayed in figure 4. Countries/regions with low population density, such as Washington (USA), South Dakota (USA) and Western Australia (AUS), had over 80% of their population that achieved very high accessibility to vaccination. By comparison, in countries/regions such as Manitoba (CAN), Ontario (CAN) and Bhutan, over 60% of the population experienced very low accessibility. This situation also occurred in areas with high population density. In countries/regions such as New York (USA), Massachusetts (USA) and Northern Ireland (UK), more than 90% of the population had very high vaccination access. Conversely, in countries such as Belgium, over 60% of the population resided in very low accessibility zones.

Vaccine accessibility also varied significantly within the borders of some countries/regions. For example, in Namibia, approximately 24% of the population experienced very low accessibility, while another 37% achieved high accessibility levels, and an additional 21% benefited from very high vaccine accessibility. This situation was also observed in other countries, such as Malaysia, the Philippines and Luxembourg. However, in some countries/regions, such as Singapore and Guangdong Province (CHN), over 90% of the population exhibited concentrated patterns, predominantly residing in moderate and high accessibility zones.

### Economic factors contributing to disparities in vaccine accessibility

**Figure 5:**
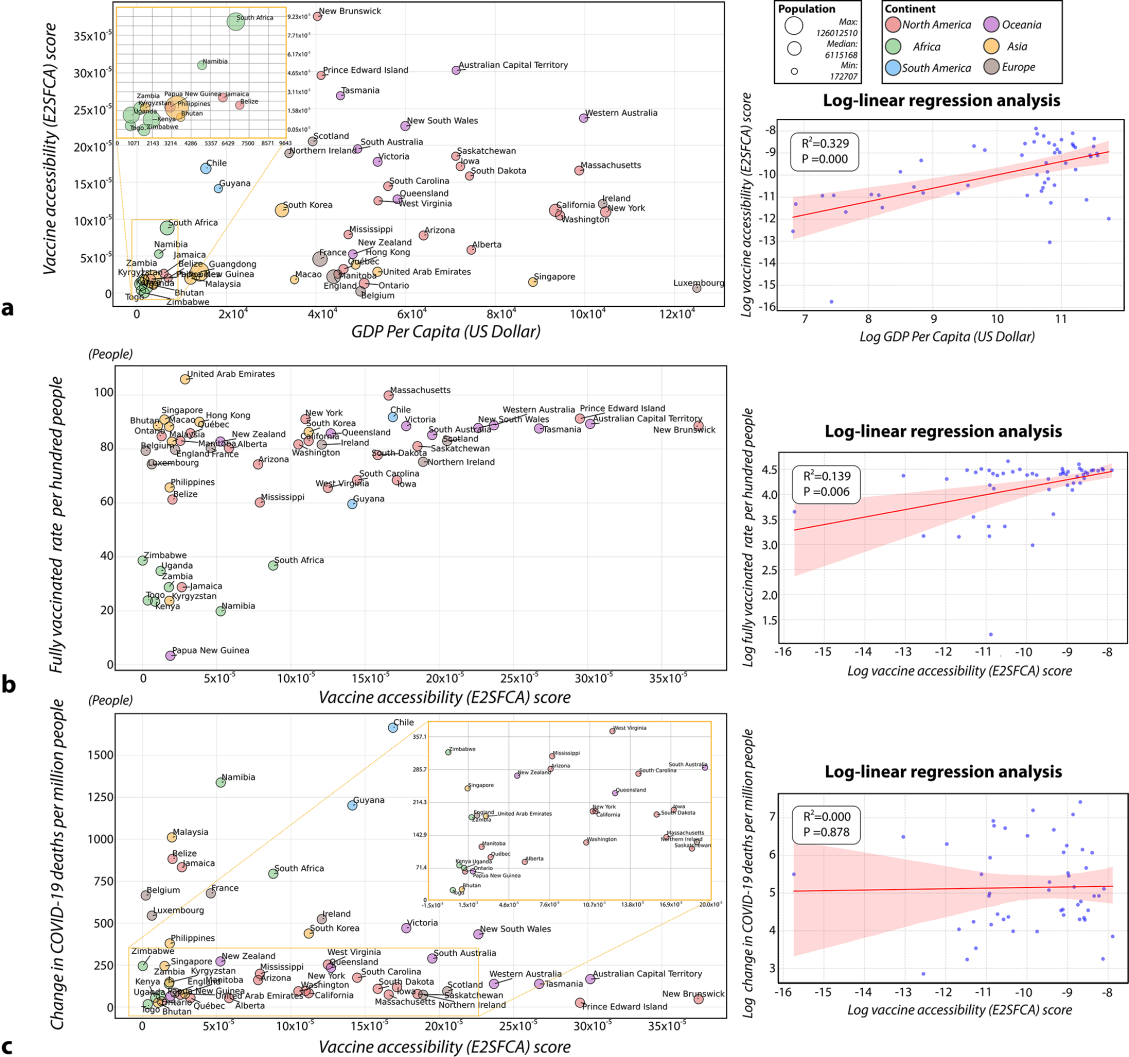
(a) Relationship between vaccine accessibility and GDP per capita (US$); (b) relationship between vaccine accessibility and full vaccination rate per hundred on 30 June 2022; (c) relationship between vaccine accessibility and the difference in total COVID-19 deaths per million from 1 December 2020 to 30 June 2022. (1) Mortality data in Guangdong Province (CHN), Hong Kong SAR (CHN) and Macao SAR (CHN) are missing due to data unavailability. (2) Dose data in Guangdong Province (CHN), Luxembourg are missing due to data unavailability. (3) Kyrgyzstan's mortality data are only available up to May 2021, so the mortality data for 1 December 2020 are treated as missing. (4) Bhutan's mortality data are only available up to 10 January 2021. We used the data on 10 January 2021, as an approximation for 1 December 2020. E2SFCA, Enhanced Two-Step Floating Catchment Area; GDP, gross domestic product.

We further explored the relationship between country/regional GDP per capita (online supplemental table S1) and COVID-19 vaccine accessibility (online supplemental table S3). In highly populated countries, vaccine accessibility tended to be lower compared with less populated countries/regions with similar GDP per capita (figure 5a). Some African and Asian countries/regions with large populations and low GDP per capita, such as Kenya, Uganda, Guangdong Province (CHN) and the Philippines, presented low in vaccine accessibility. In contrast, countries/regions including Western Australia (AUS), Prince Edward Island (CAN) and Australian Capital Territory (AUS), which have smaller populations and relatively higher GDP per capita, tend to have better vaccine accessibility. However, we also observed that countries such as Ireland, Luxembourg and New York (USA), despite their high GDP per capita and smaller populations, still suffer from low vaccine accessibility. To quantify the relationship, our log-linear regression revealed a significant positive relationship between GDP per capita and vaccine accessibility ($R^{2}$=0.323, p<0.001).

### Vaccine accessibility and the corresponding health outcomes

The relationship between vaccine accessibility and vaccination rates (online supplemental table S3) revealed that overall countries with greater vaccine accessibility tend to achieve higher vaccination rates (figure 5b). This is particularly evident in HICs such as states within Australia and the USA. In contrast, LMICs such as Zimbabwe, Kyrgyzstan and Togo typically experienced lower vaccination rates, which could be attributed to their limited geographic access to vaccines. Furthermore, several other countries, such as UAE, Singapore, Belgium and France, have lower or moderate accessibility levels, yet they maintain high vaccination rates. The log-linear regression analysis demonstrated a significant positive relationship between vaccine accessibility and vaccination rates ($R^{2}$= 0.139, p<0.01).

The association between vaccine accessibility and COVID-19 mortality showed that approximately 60% of countries/regions experienced minimal increase (less than 300 deaths increment per million population) during December 2020 and June 2022 (figure 5c). In contrast, countries/regions with lower vaccine accessibility, such as France, Belgium, Malaysia and Belize, experienced greater increase in COVID-19 mortality. Notably, Chile and Guyana surged in mortality while their vaccine access was high. The log-linear regression analysis revealed no significant relationship between vaccine accessibility and COVID-19 mortality ($R^{2}$=0.000, p>0.05).

## Discussion

The study evaluated geographic accessibility to COVID-19 vaccination sites across 54 countries/regions. To our knowledge, this research is the first to explore vaccine accessibility and corresponding health outcomes across a diverse range of HICs and LMICs. Our fine-scale analysis provides an understanding of the current strategy of disease control for individual countries/regions and further allows comparison in terms of effectiveness across countries/regions.

Our findings reveal substantial variation in geographic accessibility to vaccines both within and across the borders of countries/regions. For countries such as Belgium, Luxembourg and Bhutan, the urban centres achieved the highest accessibility, where vaccination sites are concentrated. This distribution bias led to lower accessibility in remote and rural areas. This situation was also reported by previous studies that the limited medical facilities in USA rural areas led to higher mortality rates compared with urban areas during the COVID-19 pandemic.^39 40^ For countries/regions with a high level of urbanisation, such as Guangdong Province (CHN), Singapore and Macao SAR, their accessibility levels are categorised as either moderate or high. This is primarily attributed to their advanced transportation infrastructure. Additionally, these regions have also established early warning systems and institutional infrastructure from past pandemic experiences including SARS in 2003 and H1N1 in 2009.^41^,^43^ However, only 2% of residents benefit from very high accessibility, possibly due to high population density. For future practice, it is recommended that these countries actively pursue vaccines and establish additional vaccination sites in densely populated areas to minimise competition for vaccine supplies.^44^ Our statistical analysis revealed a significant association between GDP per capita and vaccine accessibility ($R^{2}$=0.323, p<0.001), indicating that economic development substantially influences the spatial distribution of vaccination infrastructure. However, there are deviations from this general pattern. For example, Luxembourg, with its high GDP per capita in 2022, shows unexpectedly low vaccine accessibility. This may result from uneven distribution of vaccination sites, concentrated in central and southwestern regions.^45^ Furthermore, Luxembourg has adopted a voluntary healthcare approach for family physicians, rather than mandatory involvement in vaccination programmes.^46^ In contrast, New Brunswick (CAN), despite its relatively lower GDP per capita, has achieved excellent vaccine accessibility. This success is attributed to the strategic planning of temporary vaccination clinics and outreach centres in areas with high disease transmission and low immunisation rates.^47^ Therefore, effective healthcare delivery relies heavily on strategic planning and community engagement in addition to economic capacity.

Furthermore, our log-linear regression analysis demonstrated a significant relationship between vaccine accessibility and vaccination rates ($R^{2}$=0.139, p<0.01), however, with a relatively low $R^{2}$. Additionally, our analysis revealed no significant relationship between vaccine accessibility and increase in COVID-19 mortality per million people ($R^{2}$=0.000, p>0.05). This lack of correlation suggests that there is no general pattern linking vaccine accessibility to COVID-19 mortality rates. Instead, the influence of vaccine access on COVID-19 mortality rates varies by country/region, impacted by factors such as healthcare system capacity,^48 49^ demographic characteristics,^50 51^ timing of public health interventions^50^ and the effectiveness of non-pharmaceutical measures.^52 53^ Notably, vaccination rates in all countries/regions increased by at least 20% from 1 May 2021 to 30 June 2022 (online supplemental figure S1), partially attributed to global health initiatives. However, we noticed Papua New Guinea had the lowest increase in vaccination rate. This is potentially due to its widely dispersed population across islands with rugged terrain, poor weather, the tense domestic political atmosphere and communication difficulties among residents.^54^ We also found several countries maintain high vaccination rates despite having lower or moderate accessibility levels. This corresponds to previous research that HICs implemented free vaccination policies and had awareness of disease severity and vaccine effectiveness.^55^ Furthermore, it is noteworthy that the UAE had vaccination rates exceeding 100%, partly due to the reported data including vaccinations for non-residents.^55 56^

It is important to note that the mortality rates in African countries (except South Africa and Namibia) did not surge during the pandemic. This may be due to the lack of vital registration systems and testing services, leaving many deaths undetected.^57^ Additionally, we observe that island countries, such as Australia, Papua New Guinea and New Zealand, experienced notable increases in mortality (online supplemental figure S2). These situations can be attributed to strict border closures and quarantine at the onset of COVID-19 in 2020.^58^ With the emergence of virus variants such as Delta and Omicron in 2021, the number of cases and deaths in these countries rapidly surged within a short period. The situation placed immense pressure on their healthcare systems, with patients quickly occupying medical facilities. The fully occupied hospitals left no room to treat patients with other prevalent diseases in these island nations, such as hepatitis and tuberculosis, which are common burdens in Pacific Island countries.^58^

Our research presents several strengths. First, the multi-indicator framework for computing vaccine accessibility provides evaluation beyond travel time or population coverage. Our analysis offers comprehensive insights into pharmaceutical control during the COVID-19 pandemic. Second, our analysis across 54 countries/regions extends beyond traditional studies, which focused on HICs, to include both LMICs and HICs. The findings from both intercountry equity and intra-country variations provide valuable insights for both local and global policymakers for future disease control. Third, our exploratory analysis between vaccine accessibility and related health outcomes (ie, vaccination rates and COVID-19 mortality) at different stages during the pandemic further assessed the effectiveness of existing COVID-19 control strategies. Typically, the multicountry comparison provides evidence to initiate a conversation with local health officials in individual country/region to adjust vaccine allocation strategies for better disease control in future pandemics.

Several limitations exist in this research. First, data availability and resourcing barriers limited our study countries/regions. The evaluation of disease control strategy at global scale was restricted by the small sample size. Restrictions from individual countries include limited open data policy, lack of online data repositories and restricted access to department of health information. Several countries only had a picture of vaccine site lists on their social media platform, with no valid source information. Future work could extend our collaborative team members to conduct an enhanced global study.

Second, the computation of geographic accessibility only considered driving as travel mode, due to the constraints of OSRM. While car-based travel is the predominant mode of transportation in most countries/regions in our study, there are some exceptions.^59^ In places with advanced public transportation networks, such as Hong Kong SAR and Singapore, residents may choose metro, buses and light rail systems for their daily commute.^60 61^ Additionally, in several LMICs such as Namibia, Kenya and Zambia, where car ownership is low, residents often rely on multiclient taxis, minibuses and moto-taxis to reach COVID-19 vaccination sites.^62 63^ Furthermore, for island nations such as Papua New Guinea, ferry is a common transportation mode to reach vaccination sites.^64^ In these cases, our analysis potentially produced bias in accessibility estimation.

Third, while exploring the relationship between vaccine accessibility and vaccination rates or mortality, we did not account for non-spatial factors such as awareness, education and religious beliefs, which are referenced in previous studies.^614 16 17 65^,^67^ These parameters have been identified to influence vaccine willingness and acceptance. This limitation may lead to exceptions in our results, where regions with high or moderate accessibility levels continue to experience lower vaccination rates.

Fourth, we lacked information on vaccine inventory and daily appointment capacities at each vaccination site. It is particularly critical because the daily capacity, which is a key factor in accessibility evaluations,^68^ varies widely across different vaccination facilities. For example, during COVID-19 vaccinations, the daily capacity for an 8-hour clinic ranged from 500 doses at smaller vaccination hubs to 1400 doses at larger ones.^69^ Similarly, general practitioners administered vaccines ranging from roughly 100 to almost 300 doses per day depending on the size of the practice.^69^ The lack of capacity information may lead to overestimating vaccine accessibility, particularly when pharmaceutical supplies or appointment availability are constrained.

Our findings suggest potential strategies for future pandemics. First, we suggest that global health organisations enhance international cooperation to ensure equitable distribution of vaccines, especially among LMICs. This approach would reduce mortality worldwide and prevent recurring waves of the pandemic.^70 71^ Second, we recommend investments by public health authorities in health infrastructure and mobile vaccine clinics, particularly in areas with low vaccine accessibility. This would potentially facilitate the equitable distribution of vaccines, global vaccination capacity and vaccine uptake rates.^72^ Additionally, in response to variations in vaccine accessibility within a country, more refined public health strategies should be developed to ensure all residents can access vaccinations promptly and equitably.^40 73^ In summary, this research has contributed to alleviating global and local inequalities in vaccine accessibility and has provided insights for the control of future pandemics.

## supplementary material

10.1136/bmjgh-2024-017761 online supplemental file 1

## Data Availability

All data relevant to the study are included in the article or uploaded as supplementary information.
