# Supplementary material for: Evaluating geographic accessibility to COVID-19 vaccination across 54 countries/regions
Source: BMJ Glob Health. 2025 Feb 19;10(2):e017761. doi: 10.1136/bmjgh-2024-017761 (PMC11840912; doi:10.1136/bmjgh-2024-017761)
Supplement: online supplemental file 1 [file bmjgh-10-2-s001.pdf]

Supplementary Appendix

Content

Supplementary Data Sources 1
Source 1. Extraction of population data at a 1 km² resolution 1
Source 2. Sources of vaccination site addresses for each country/region 2
Source 3. Sources of GDP per capita for each country/region 5
Source 4. Source of population in 2022 for each country/region 5
Source 5. Sources of COVID-19 vaccine dose for each country/region 6
Source 6. Sources of COVID-19 mortality data for each country/region 7
Supplementary Method 8
Method S1. Google Maps Geocoding API 8
Method S2. OSRM Methodology for Aligning OD Points and Driving Time Computation... 9
Supplementary Results 11
Table S3. Vaccine accessibility, vaccination rate per hundred people, and total deaths per million as of December 1, 2020, May 1, 2021, and June 30, 2022 14
Figure S2. Distribution of vaccine accessibility and the difference in total COVID-19 deaths per million from December 1, 2020, to June 30, 2022, in different countries/regions. 17

Supplementary Data Sources

Source 1. Extraction of population data at a 1 km² resolution

(1) The population data utilized in this study was sourced from Bondarenko M.'s Global 1km Population dataset for individual countries, 2020. This dataset is available at WorldPop, under the listing 'Population Counts / Unconstrained individual countries 2000-2020 UN adjusted (1km resolution)' and can be accessed through the following link: https://hub.worldpop.org/geodata/listing?id=75.

(2) For the SHP data of individual countries' boundaries, we downloaded it from geoBoundaries (https://www.geoboundaries.org/globalDownloads.html).

(3) For state-level boundary SHP data, we primarily downloaded it from the official government websites of the respective countries.

- United State boundary data:
http://www.census.gov/geographies/mapping-files/time-series/geo/carto-boundary-file.html
- Canada boundary data:
https://www12.statcan.gc.ca/census-recensement/2021/geo/sip-pis/boundary-limites/index2021-eng.cfm?year=21
- Australia boundary data:
https://www.abs.gov.au/statistics/standards/australian-statistical-geography-standard-asgs-edition-3/jul2021-jun2026/access-and-downloads/digital-boundary-files
- United Kingdom boundary data:
https://www.data.gov.uk/dataset/50fb9e41-01d4-4e12-b5a2-c9add02470a8/local-authority-districts-december-2021-boundaries-uk-buc

- Guangdong 2022 population data:

University of Michigan. China Data Center. Beijing Hua tong ren shi chang xin xi you xian ze ren gong si. (2005). County boundaries: Guangdong Sheng Province, China, 2000. [Shapefile]. Beijing Hua tong ren shi chang xin xi you xian ze ren gong si. Retrieved from https://hgl.harvard.edu/catalog/stanford-zd752vr7832

Source 2. Sources of vaccination site addresses for each country/region

Data on vaccination sites were mainly sourced from the health department websites of the countries. Due to the unavailability of vaccine supply data for all countries, we assumed an equal and unlimited supply of vaccines at each site. The original vaccination site data, which included detailed addresses and postal codes such as "National Road, Población, 8M8V+PGC, Luba, 2813 Abra, Philippines," were converted into geographic coordinates (longitude and latitude) using the WGS-84 coordinate system via Google's Geocoding API[1].

Reference:

1. Google. Geocoding API of Google Maps Platform. 2021. https://developers.google.com/maps/documentation/geocoding/start

Asia:

- Guangdong (CHN) (Accessed on 2023-08-27):

http://cdcp.gd.gov.cn/jkjy/jkzt/ymymy/myjzzcjjswj/content/post_3442967.html

- Macao SAR (Accessed on 2023-08-27):

https://eservice.ssm.gov.mo/covidvacbook/Booking/Waitingstatus?lang=ch

- Hong Kong SAR (Accessed on 2021-05-01):
  - Map of vaccination sites

    https://www.map.gov.hk/gm/map/s/m/communityvaccinationservicesofcovid-19?lg=en

    [The website has been inactive since October 2023.]
  - Vaccination sites (Community vaccination centres) can be downloaded at geodata.gov.uk

    https://geodata.gov.hk/gs/datasets?s=vaccination

    [The website has been inactive since October 2023.]
- Malaysia (Accessed on 2021-06-17):

https://www.vaksincovid.gov.my/en/ppv/

[The website has been inactive since October 2023.]

- Singapore (Accessed on 2021-06-17):

https://www.vaccine.gov.sg/locations-vcs

[The website has been inactive since October 2023.]

- South Korea (Accessed on 2023-09-07):

Korean Disease Control and Prevention Agency (KDCA).

https://ncvr.kdca.go.kr/cobk/rsrv/web/getVcnRsrvMdinLst.do

[The website has been inactive since October 2023.]

- Philippines (Accessed on 2023-08-27):

https://doh.gov.ph/covid-19-vaccination-sites

[The website has been inactive since October 2023.]

- Kyrgyzstan (Accessed on 2023-09-07):

https://vc.emed.gov.kg/lpus/IndexAllPeople

[The website has been inactive since October 2023.]

- Bhutan (Accessed on 2023-09-07):

https://www.moh.gov.bt/vaccination-post-for-second-dose-vaccination-campaign-in-thimphu-thromde/

[The website has been inactive since October 2023.]

- United Arab Emirates (Accessed on 2023-09-07):

https://covid19.ncema.gov.ae/en/vaccinecenters/list

Oceania:

- Papua New Guinea (Accessed on 2023-08-27):

https://covid19.info.gov.pg/covid-19-vaccination-sites-in-papua-new-guinea/

- New Zealand (Accessed on 2023-08-27):

https://www.healthpoint.co.nz/covid-19-vaccination/?options=walk-in

Vaccination location map | Book My Vaccine | Te Whatu Ora – Health New Zealand

- Australia (Accessed on 2023-08-27):

Where you can get vaccinated | Australian Government Department of Health and Aged Care

North America:

- United States (Accessed on 2021-06-17):

https://www.vaccines.gov/

- Canada (Accessed on 2021-06-17):

https://www.canada.ca/en/public-health/services/diseases/coronavirus-disease-covid-19/vaccines/how-vaccinated.html#a1

- Jamaica (Accessed on 2023-09-07):

https://vaccination.moh.gov.jm/vaccination-sites/

- Belize (Accessed on 2023-09-07):

https://www.myvaccine.bz/vaccination-sites/

[The website has been inactive since October 2023.]

South America:

- Guyana (Accessed on 2023-09-07):

https://www.health.gov.gy/index.php/vaccination-sites

[The website has been inactive since October 2023.]

- Chile (Accessed on 2021-06-17):

https://www.gob.cl/yomevacuno/vacunatorios/

[The website has been inactive since October 2023.]

Europe：

- Belgium (Accessed on 2023-08-27):

https://coronavirus.brussels/nl/vaccinatie-covid-menu/waar-kan-ik-me-laten-vaccineren/#Interactieve_kaart_van_de_verschillende_Covid-vaccinatiepunten_in_het_Brussels_Gewest

[The website has been inactive since October 2023. It links to another webpage on COVID-19.]

- Ireland (Accessed on 2023-08-27):

https://www2.hse.ie/services/pharmacies-flu-and-covid-vaccines/?service_area=Carlow

- Luxembourg (Accessed on 2023-09-07):

https://covid19.public.lu/en/vaccination/centre-vaccination.html

[The website has been inactive since October 2023. It links to another webpage on COVID-19]

- France (Accessed on 2021-06-15):

https://www.data.gouv.fr/en/datasets/lieux-de-vaccination-contre-la-covid-19/

- United Kingdom
  - England: (Accessed on 2021-06-15):

    The list of vaccination sites is accessible to the guidance of NHS publication:

    https://www.england.nhs.uk/coronavirus/publication/vaccination-sites/

    [The website has been inactive since October 2023.]
  - Scotland: (Accessed on 2021-01-05):

    The list of vaccination sites is accessible at gov.scot

    https://www.gov.scot/publications/coronavirus-covid-19-daily-data-for-scotland/

    [The website is still working, but it seems that the vaccination sites are not available.]
  - Northern Ireland: (Accessed on 2021-06-15):

    The list of vaccination sites (community pharmacies) is from Health and Social Care Northern Ireland (HSCNI):

    http://www.healthandcareni.net/pharmacy_rota/Covid_Vaccination_Pharmacies.html

    [The website has been inactive since October 2023.]

    The list of vaccinations sites (GP) is from HSCNI

    https://hscbusiness.hscni.net/services/1816.htm

Africa:

- South Africa (Accessed on 2023-09-07):

https://ckan.africadatahub.org/dataset/ba851022-05b8-4999-a2ba-6c8627be5523/resource/93f60e80-a688-4b01-9994-6eb561bba9ce/download/za-vaccination-sites.csv

- Zambia (Accessed on 2023-09-07):

https://www.africageoportal.com/datasets/e9b3fd9902e544e9b95f9b72bc1e8804_0/explore?location=-13.389567%2C29.266455%2C6.30

[The website has been inactive since October 2023.]

- Zimbabwe (Accessed on 2023-09-07):

https://www.herald.co.zw/govt-increases-covid-19-vaccination-sites/

- Uganda (Accessed on 2023-09-07):

https://www.uvri.go.ug/news/approved-covid-19-vaccination-sites-uganda

- Togo (Accessed on 2023-09-07):

https://vaccin.covid19.gouv.tg/sites/

- Namibia (Accessed on 2023-09-07):

https://nhp.com.na/wp-content/uploads/2021/08/COVID-Nationwide-vaccination-Sites.pdf

[The website has been inactive since October 2023.]

- Kenya (Accessed on 2023-09-07):

http://www.minet.com/wp-content/uploads/2021/08/MOH-Approved-COVID-19-Vaccination-Centres_August-2021.pdf

Source 3. Sources of GDP per capita for each country/region

GDP per capita, (current US$) in 2022: GDP per capita, expressed in current US dollars for the year 2022, indicates the average economic output per person in a country, converted to the value of US dollars for that specific year.

Current US dollar fluctuate daily based on exchange rates. We accessed the data on July 8, 2024, and collected all data on the same day for a cross-sectional comparison. For the dataset originally expressed in their local currencies, their figures are converted to US dollars using the exchange rate as of July 9, 2024.

(1) The Country Level Economic Data were accessed from World Bank databases.
https://databank.worldbank.org/source/world-development-indicators
(2) State-level Economic data were sourced from the official government websites of the respective countries.

- United States state-level data:
https://www.bea.gov/sites/default/files/2023-03/stgdppi4q22-a2022.pdf
- United Kingdom country-level data:
https://www.ons.gov.uk/economy/grossdomesticproductgdp/bulletins/regionaleconomicactivitybygrossdomesticproductuk/latest
- Canada provincial-level date:
https://www150.statcan.gc.ca/t1/tbl1/en/cv.action?pid=3610022101
- Australia state-level data:
https://www.abs.gov.au/statistics/economy/national-accounts/australian-national-accounts-state-accounts/latest-release
- Guangdong data:
https://data.stats.gov.cn/english/easyquery.htm?cn=E0102
https://data.stats.gov.cn/english/easyquery.htm?cn=E0103

* The data for Guangdong uses the per capita gross domestic product (GDP) of Guangdong province, China for the year 2022, expressed in yuan. This figure is converted to US dollars using the exchange rate as of July 9, 2024.

Source 4. Source of population in 2022 for each country/region

(1) The Country Level population data were accessed from World Bank databases.
https://databank.worldbank.org/reports.aspx?source=2&series=SP.POP.TOTL&country=
(2) State-level population data were sourced from the official government websites of the respective countries.

- United State 2022 population data:
https://www.census.gov/data/tables/time-series/demo/popest/2020s-state-total.html#v2022
- Canada 2022 population data:

https://worldpopulationreview.com/canadian-provinces

- Australia 2022 population data:

https://population.gov.au/data-and-forecasts/key-data-releases/national-state-and-territory-population-march-2022

- United Kingdom 2022 population data:

https://www.ons.gov.uk/peoplepopulationandcommunity/populationandmigration/populationestimates/bulletins/annualmidyearpopulationestimates/mid2022

- Guangdong 2022 population data:

https://research.hktdc.com/en/data-and-profiles/mcpc/provinces/guangdong/guangzhou

Source 5. Sources of COVID-19 vaccine dose for each country/region

People vaccinated per hundred: People vaccinated per 100 people in the total population of the country/region indicates those who have received a complete COVID-19 primary vaccine series. This includes having received the number of doses of a COVID-19 vaccine corresponding to that product's primary series, as per its product-specific use authorization in the country where it was administered. The number of doses constituting a primary series varies by product and by country. For most vaccine products in most countries, a complete primary series consists of two doses.

(1) Country-level vaccine dose data was collected from WHO
https://data.who.int/dashboards/covid19/data?n=o

(2) State-level vaccine dose data were sourced from the health department websites of the respective countries.

- United States state-level data:

It is collected by 'Our World in Data' Project[3]. The raw data is updated daily by
https://covid.cdc.gov/covid-data-tracker/#vaccine-delivery-coverage

- United Kingdom state-level data:

https://ukhsa-dashboard.data.gov.uk/topics/covid-19#vaccinations
[The website has been inactive since October 2023.]

- Canada state-level data:

https://health-infobase.canada.ca/covid-19/vaccination-coverage/

- Australia state-level data:

https://www.health.gov.au/resources/collections/covid-19-vaccination-rollout-update?language=en#march-2024

Reference:

1. Google. Geocoding API of Google Maps Platform. 2021. https://developers.google.com/maps/documentation/geocoding/start
2. World Health Organization. WHO Coronavirus (COVID-19) dashboard > Vaccines [Dashboard]. 2023. https://data.who.int/dashboards/covid19/vaccines.
3. Edouard Mathieu HR, Lucas Rodés-Guirao , Cameron Appel , Charlie Giattino and Joe Hasell ,Bobbie Macdonald , Saloni Dattani , Diana Beltekian , Esteban Ortiz-Ospina , Max Roser. Coronavirus Pandemic (COVID-19). Our World in Data 2020.

Source 6. Sources of COVID-19 mortality data for each country/region

Total death per million: The total number of deaths per million people due to COVID-19 helps to understand and compare the severity of the pandemic across different countries or regions.

(1) Country-level COVID-19 Mortality data was collected from WHO
https://www.who.int/data/data-collection-tools/who-mortality-database

- The definition of COVID-19 death is guided by
https://www.who.int/docs/default-source/classification/icd/covid-19/guidelines-cause-of-death-covid-19-20200420-en.pdf
- "A death due to COVID-19 is defined for surveillance purposes as a death resulting from a clinically compatible illness, **in a probable or confirmed COVID-19 case**, unless there is a clear alternative cause of death that cannot be related to COVID disease (e.g. trauma). **There should be no period of complete recovery from COVID-19 between illness and death**.

Bhutan 2020.12.1 data from 2021-01-10

(2) State-level vaccine dose data were sourced from the health department websites of the respective countries.

- United States state-level data:
https://covid.cdc.gov/covid-data-tracker/#trends_totaldeaths_totaldeathrateaa_04
  - The definition of COVID-19 death is guided by
https://www.who.int/docs/default-source/classification/icd/covid-19/guidelines-cause-of-death-covid-19-20200420-en.pdf
    - Source: Provisional Deaths from the CDC's National Center for Health Statistics (NCHS) National Vital Statistics System (NVSS).
    - Provisional data are non-final counts of deaths based on the flow of mortality data in NVSS. **Deaths include those with COVID-19, coded to ICD–10 code U07.1, as an underlying or contributing cause of death on the death certificate.** Death data are displayed by date of death (event). This is a change from the surveillance data used previously in COVID Data Tracker which were displayed by date of report.
    - **Rates were calculated as the number of provisional deaths occurring in the specified week/month divided by the population estimated as of July 1, 2021, multiplied by 100,000.** Population estimates are from the Blended Base produced by the U.S. Census Bureau in lieu of the April 1, 2020 decennial population count.

(see https://www2.census.gov/programs-surveys/popest/technical-documentation/methodology/2020-2021/methods-statement-v2021.pdf).

- United Kingdom state-level data:

UK Health Security Agency (UKHSA) data dashboard
https://coronavirus.data.gov.uk/details/download
  - The definition of COVID-19 death is guided by
https://assets.publishing.service.gov.uk/media/61fb93118fa8f53893357fc7/UKHSA-technical-summary-update-February-2022.pdf
  - There are 2 measures of a death in a person with COVID-19 in England, one measure to reflect

current trends and one comprehensive measure that incorporates death certificate data:

- ◆ (1) A death in a person with a positive SARS-CoV-2 test and died within (equal to or less than) **28 days of the first positive specimen date** of the most recent infection.
- ◆ (2) A death in a person with a positive SARS-CoV-2 test and either died within 60 days of the first specimen date of the most recent infection or died more than **60 days after the first specimen date of the most recent infection,** only if COVID-19 is mentioned on the death certificate.

- ● Canada state-level data:

the Public Health Agency of Canada (PHAC)
https://health-infobase.canada.ca/covid-19/current-situation.html?stat=rate&measure=deaths_total&map=pt#a2

- ■ The definition of COVID-19 death is guided by

https://www.canada.ca/en/public-health/services/diseases/2019-novel-coronavirus-infection/health-professionals/national-case-definition.html

- ◆ **A probable or confirmed COVID-19 case** whose death **resulted from a clinically compatible illness, unless there is a clear alternative cause of death identified** (e.g., trauma, poisoning, drug overdose).
- ◆ A Medical Officer of Health, relevant public health authority, coroner or medical examiner may use their discretion when determining if a death was due to COVID-19, and their judgement will supersede the above-mentioned criteria.
- ◆ A death due to COVID-19 may be attributed **when COVID-19 is the cause of death or is a contributing factor**.

- ● Australia state-level data:

Department of Health and Aged Care (DHAC)
https://www.health.gov.au/topics/covid-19/reporting#covid19-associated-deaths

- ■ The definition of COVID-19 death is guided by

https://www.health.gov.au/sites/default/files/documents/2022/10/coronavirus-covid-19-cdna-national-guidelines-for-public-health-units.pdf
[The website has been inactive since October 2023.]

- ◆ The definition in the document is the same as WHO's definition of a COVID-19 death.

Supplementary Method

Method S1. Google Maps Geocoding API

In our research approach, as outlined in the research framework (Figure 1(b)), we initially processed address information by converting it into geographic coordinates. This conversion was accomplished

using the Google Maps Geocoding API, which employs the World Geodetic System 1984 (WGS-84) as its coordinate system. This system is a global reference system for geography and navigation, providing accurate locations on the earth's surface.

The Google Maps Geocoding API is a robust tool that translates addresses into geocodes (latitude and longitude coordinates), enabling precise location mapping. This method is particularly effective for applications requiring spatial analysis or geographic data integration. By utilizing this API, we were able to ensure that our spatial data was both accurate and consistent, facilitating subsequent analysis stages in our study. This process is critical for ensuring that the geographic data aligns with global standards and allows for reliable comparisons and assessments in geographic research.

Reference: Google. Geocoding API of Google Maps Platform. 2021. https://developers.google.com/maps/documentation/geocoding/start.

Method S2. OSRM Methodology for Aligning OD Points and Driving Time Computation

We utilized the Open Source Routing Machine (OSRM) to align origin-destination (OD) points with the nearest street network, which is essential for accurate driving time calculations.

OSRM Alignment Process:

(1) We input origin-destination (OD) pairs between population centroids and vaccination sites directly into OSRM.
(2) OSRM matches these points to the closest roads, ensuring they are positioned on valid, drivable routes.
(3) The coordinates of OD points are adjusted to align precisely with the road network.

Driving Time Computation:

(1) Route Calculation: OSRM computes optimal driving routes between points, considering road type, distance, and traffic conditions.
(2) Speed Limit Applicatio: Speed limits for different road types are incorporated into the route calculations, ensuring realistic driving time estimates.

OSRM Configuration and Utilization:

(1) OSRM was set up to prioritize efficiency, accounting for fastest travel time.
(2) This tool facilitated our analysis by providing accurate travel time estimates for various planning scenarios.

Reference: OSRM P. OSRM API Documentation (Version 5.24.0) 2021. https://project-osrm.org/docs/v5.24.0/api/#services.

Supplementary Results

Table S1. Population and GDP per capita data used in the paper

Continent	Province	GDP per capita	Population
North America	Alberta	74663.00	4601314
	Saskatchewan	71195.00	1205119
	Manitoba	44893.00	1420228
	Ontario	50755.00	15262660
	Québec	46134.00	8751352
	New Brunswick	40309.00	820786
	Prince Edward Island	41124.00	172707
	Massachusetts	98750.00	6982740
	Arizona	64010.00	7365684
	Washington	94470.00	7784477
	South Dakota	74268.00	909869
	Iowa	72221.00	3199693
	California	93460.00	39040616
	Mississippi	47190.00	2938928
	South Carolina	56066.00	5282955
	West Virginia	53852.00	1774035
	New York	104660.00	19673200
	Belize	6984.22	405272
	Jamaica	6047.22	2827377
Africa	Togo	923.24	8848699
	Uganda	964.35	47249585
	Kenya	2099.30	54027487
	Zambia	1456.90	20017675
	Zimbabwe	1676.82	16320537
	Namibia	4895.68	2567012
	South Africa	6766.48	59893885
South America	Chile	15411.17	19603733
	Guyana	18199.47	808726
Oceania	Western Australia	99736.00	2773435
	Queensland	58172.00	5296098
	New South Wales	59931.00	8130115
	Victoria	53756.00	6593314

	South Australia	49292.00	1815485
	Tasmania	45451.00	571165
	Australian Capital Territory	71282.00	455869
	Papua New Guinea	3115.92	10142619
	New Zealand	48216.51	5124100
Asia	Guangdong	14020.85	126012510
	Macao	35192.53	695168
	Hong Kong	48828.12	7346100
	Bhutan	3704.02	782455
	Kyrgyzstan	1739.80	6974900
	Malaysia	11993.19	33938221
	Singapore	88428.70	5637022
	South Korea	32394.68	51628117
	Philippines	3499.11	115559009
	United Arab Emirates	53707.98	9441129
Europe	Scotland	39242.00	5448000
	England	43931.00	57106000
	Northern Ireland	33974.00	1911000
	Ireland	103983.29	5127170
	Belgium	49942.09	11685814
	Luxembourg	125006.02	653103
	France	40886.25	67971311

Table S2. National income levels, population coverage by travel time, and median travel time to the nearest vaccination sites

**Continent**	**Country/Region**	**Income level**	**Population coverage (%) with**			**Median travel time (minutes) to the nearest vaccination site**
			**15min**	**30min**	**60min**	
North America	Alberta	HIC	0.89	0.94	0.97	14.49
	Saskatchewan	HIC	0.80	0.85	0.95	24.87
	Manitoba	HIC	0.05	0.05	0.07	167.28
	Ontario	HIC	0.45	0.83	0.93	33.76
	Québec	HIC	0.61	0.87	0.93	24.65
	New Brunswick	HIC	0.86	0.96	1.00	10.65
	Prince Edward Island	HIC	0.89	1.00	1.00	11.11
	Massachusetts	HIC	0.99	1.00	1.00	6.89

	Arizona	HIC	0.92	0.97	0.99	10.08
	Washington	HIC	0.92	0.98	1.00	11.71
	South Dakota	HIC	0.84	0.92	0.97	11.53
	Iowa	HIC	0.90	0.99	1.00	9.25
	California	HIC	0.97	0.99	1.00	7.93
	Mississippi	HIC	0.78	0.96	1.00	15.62
	South Carolina	HIC	0.91	1.00	1.00	11.13
	West Virginia	HIC	0.82	0.97	1.00	13.23
	New York	HIC	0.97	1.00	1.00	9.65
	Belize	UMIC	0.44	0.68	0.91	32.99
	Jamaica	UMIC	0.76	0.97	1.00	16.61
Africa	Togo	LIC	0.54	0.73	0.93	37.26
	Uganda	LIC	0.49	0.84	0.99	24.05
	Kenya	LMIC	0.60	0.81	0.91	41.38
	Zambia	LMIC	0.49	0.67	0.87	42.82
	Zimbabwe	LMIC	0.13	0.21	0.27	167.11
	Namibia	UMIC	0.67	0.82	0.95	22.18
	South Africa	UMIC	0.81	0.93	0.99	16.42
South America	Chile	HIC	0.90	0.97	0.99	13.18
	Guyana	HIC	0.81	0.89	0.95	11.84
Oceania	Western Australia	HIC	0.97	0.98	0.99	6.76
	Queensland	HIC	0.92	0.96	0.98	10.43
	New South Wales	HIC	0.90	0.96	0.99	9.58
	Victoria	HIC	0.97	0.99	1.00	7.62
	South Australia	HIC	0.93	0.97	0.99	7.79
	Tasmania	HIC	0.93	0.98	1.00	3.31
	Australian Capital Territory	HIC	1.00	1.00	1.00	3.27
	Papua New Guinea	LMIC	0.28	0.54	0.79	47.11
	New Zealand	HIC	0.90	0.96	0.99	13.21
Asia	Guangdong	UMIC	0.97	1.00	1.00	8.73
	Macao	HIC	1.00	1.00	1.00	2.87
	Hong Kong	HIC	0.99	1.00	1.00	7.99
	Bhutan	LMIC	0.12	0.17	0.27	240.15
	Kyrgyzstan	LMIC	0.39	0.47	0.57	124.50
	Malaysia	UMIC	0.78	0.93	0.98	24.81
	Singapore	HIC	1.00	1.00	1.00	4.50
	South Korea	HIC	0.98	1.00	1.00	6.06
	Philippines	LMIC	0.68	0.86	0.96	23.11
	United Arab Emirates	HIC	0.26	0.72	0.98	30.53
Europe	Scotland	HIC	0.99	1.00	1.00	6.06
	England	HIC	0.91	1.00	1.00	11.46
	Northern Ireland	HIC	0.99	1.00	1.00	6.72

	Ireland	HIC	0.94	0.99	1.00	9.57
	Belgium	HIC	0.33	0.66	0.96	33.80
	Luxembourg	HIC	0.65	0.85	0.99	25.91
	France	HIC	0.88	1.00	1.00	11.82

Table S3. Vaccine accessibility, vaccination rate per hundred people, and total deaths per million as of December 1, 2020, May 1, 2021, and June 30, 2022

**Continent**	**Country/ Region**	**Accessibility level (E2SFCA Score)**	**people vaccinated per hundred**		**Total death per million**		
			**2021-5-1**	**2022-6-30**	**2020-12-1**	**2021-5-1**	**2022-6-30**
North America	Alberta	0.0000583	29.52	80.34	13.11	44.77	98.35
	Saskatchewan	0.0001853	34.52	81.04	4.0757	40.86	118.26
	Manitoba	0.0000255	29.89	83.04	22.6157	67.08	140.62
	Ontario	0.0000129	33.61	84.76	24.1486	53.98	88.42
	Québec	0.0000323	36.47	85.84	75.8886	117.23	171.37
	New Brunswick	0.0003748	31.23	88.64	0.84	4.31	51.26
	Prince Edward Island	0.0002948	28.44	91.27	0	0	25.89
	Massachusetts	0.0001659	56.72	99.83	126.286	191	265.33
	Arizona	0.0000779	40.97	74.29	86.9	213	373.87
	Washington	0.0001050	46.82	81.71	36.1571	69.4	163.47
	South Dakota	0.0001585	44.96	77.76	138.4286	218	326.56
	Iowa	0.0001715	44.31	68.45	110.571	190	308.7
	California	0.0001120	49.21	82.99	52.7571	165	248.2
	Mississippi	0.0000791	31.11	60.17	136	252	450.59
	South Carolina	0.0001446	36.74	68.46	89.3857	191	366.14
	West Virginia	0.0001249	35.9	65.58	49.5714	165	418.49

	New York	0.0001098	48.04	91.1	175.286	274	370.33
	Belize	0.0000202	10.75	61.28	347.903	792.04	1675.36
	Jamaica	0.0000265	5.01	28.76	88.775	267.39	1101.02
Africa	Togo	0.0000036	2.21	23.84	7.233	13.67	31.08
	Uganda	0.0000122	0.72	34.79	4.783	7.18	76.61
	Kenya	0.0000085	1.61	23.4	26.746	48.18	104.6
	Zambia	0.0000178	0.19	28.76	17.834	62.2	199.97
	Zimbabwe	0.0000001	2.62	38.62	16.85	95.34	340
	Namibia	0.0000527	0.91	19.83	62.719	243.47	1581.99
	South Africa	0.0000881	0.53	36.78	357.95	903.68	1698.52
South America	Chile	0.0001688	41.69	91.84	781.586	1313.12	2978.26
	Guyana	0.0001414	16.98	59.58	184.24	342.51	1544.4
Oceania	Western Australia	0.0002368	8.3	88.8	3.25	3.25	141.34
	Queensland	0.0001269	8.86	85.8	1.13	1.32	235.65
	New South Wales	0.0002263	8.21	87.8	6.52	6.64	440.46
	Victoria	0.0001777	8.28	88.5	124.37	124.37	594.85
	South Australia	0.0001952	9.11	85.2	2.2	2.2	291.93
	Tasmania	0.0002673	13.47	87.6	22.76	22.76	161.07
	Australian Capital Territory	0.0003017	11.93	89.3	6.58	6.58	173.3
	Papua New Guinea	0.0000188	0.02	3.334	0.69	0.89	65.27
	New Zealand	0.0000525	3.96	82.77	4.821	5.01	277.13
Asia	Guangdong	0.0000285	No data	No data	No data	No data	No data
	Macao	0.0000180	10.03	88.28	No data	No data	No data
	Hong Kong	0.0000384	12.5	90.06	No data	No data	No data
	Bhutan	0.0000104	61.41	88.566	1.278	1.28	26.84
	Kyrgyzstan	0.0000181	0.44	23.7	No data	6.79	154.44
	Malaysia	0.0000198	2.67	82.72	10.431	42.02	1053.5

	Singapore	0.0000148	28.82	90.93	5.145	5.15	249.96
	South Korea	0.0001120	6.43	86.3	10.093	34.99	473.41
	Philippines	0.0000183	1.43	65.72	72.11	144.29	523.7
	United Arab Emirates	0.0000287	57.26	105.83	60.268	166.19	244.78
Europe	Scotland	0.0002054	51.55	82.97	68.8	130.5	225
	England	0.0000220	51.1	79.71	91.5	191.7	277.4
	Northern Ireland	0.0001891	49.46	75.25	53.3	108.5	181.7
	Ireland	0.0001208	24.22	81.63	411.896	986.44	1510.22
	Belgium	0.0000022	27.03	79.34	1431.461	2067.96	2733.2
	Luxembourg	0.0000063	22.92	74.32	305.744	813.77	1358.86
	France	0.0000460	23.94	80.41	762.488	1642.96	2320.95

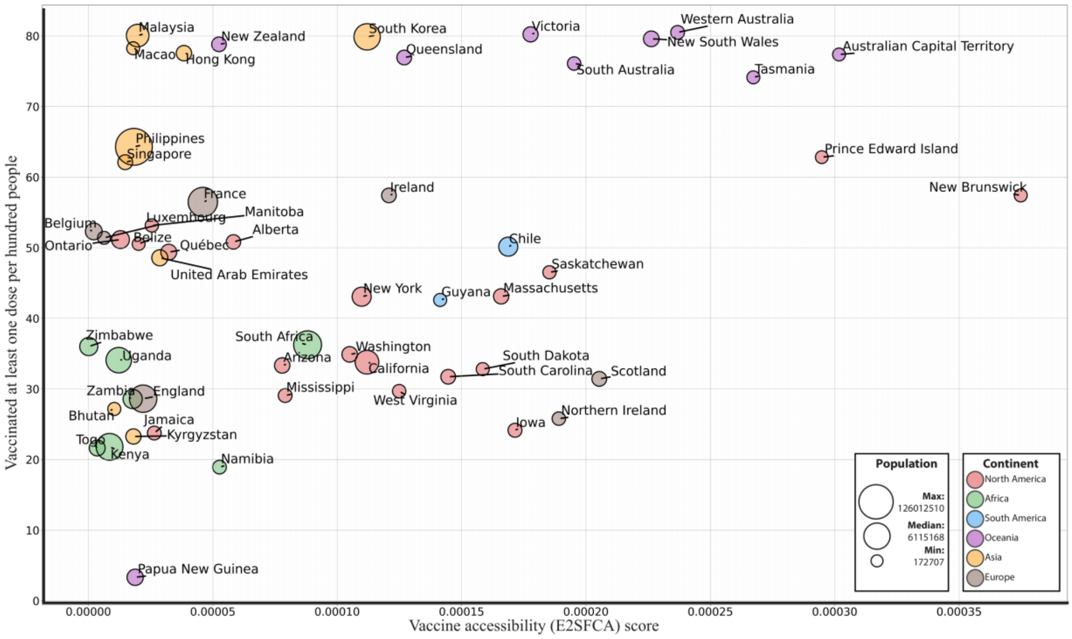

Figure S1. Distribution of vaccine accessibility and the difference of people vaccinated per hundred from May 1, 2021, to June 30, 2022, in different countries/regions.

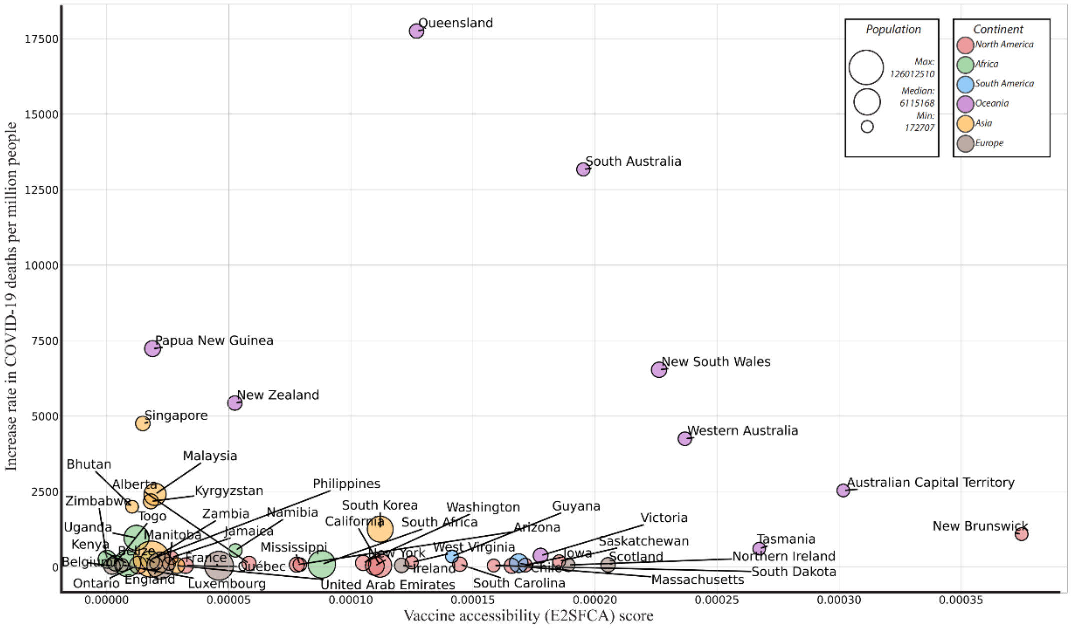

Figure S2. Distribution of vaccine accessibility and the difference in total COVID-19 deaths per million from December 1, 2020, to June 30, 2022, in different countries/regions.
